# Bromide Dose in Dogs With Epilepsy Living Close to Coastal Areas and Living More Inland: A Retrospective Observational Study

**DOI:** 10.3389/fvets.2022.906288

**Published:** 2022-05-13

**Authors:** Esther A. Lichtenauer, Bas Evers, Jan van den Broek, Paul J. J. Mandigers

**Affiliations:** ^1^Evidensia Dierenziekenhuizen, Arnhem, Netherlands; ^2^Department of Clinical Sciences, Faculty of Veterinary Medicine, Utrecht University, Utrecht, Netherlands; ^3^Centre for Biostatistics, Faculty of Veterinary Medicine, Utrecht University, Utrecht, Netherlands

**Keywords:** epilepsy, potassium bromide, anticonvulsant, salt, aerosols, sea

## Abstract

Potassium bromide is a frequently used antiseizure medication with a half-life time of over 25 days. Oral intake of sodium chloride as well as renal function influence this half-life time and may have an influence on the needed dose to *get proper serum* levels. The hypothesis is that dogs living close to coastal areas require a greater potassium bromide dose than dogs living more inland. The main study objective was to determine the relationship between bromide dose, serum bromide concentration, treatment duration, type of food, concurrent therapies and the proximity of the dog's residency to a coastal area. A retrospective cross-sectional study was performed. A total of 658 bromide serum measurements were retrieved from the veterinary faculty's laboratory archive, Utrecht University, The Netherlands. Information on the bromide dose, renal function, treatment period, type of food, concurrent therapies and residence was obtained retrospectively from owners of all dogs using a postal survey. A dataset of 220 unique study units was created. The dogs were grouped based on their residence (proximity to the coast > or <50 km). Differences between the groups of dogs regarding bromide dose, serum bromide concentration, treatment duration, type of food and concurrent therapies were analyzed to evaluate the effect of residence on bromide dose and serum concentration.

**Results:**

Although not statistically significant there is a trend that dogs living in close proximity to the sea may require a higher dose of potassium bromide to maintain therapeutic concentrations compared to dogs living more inlands. Additional studies are needed to further explore this observation.

## Introduction

Bromide (most often administered as potassium bromide) was the first drug used for the treatment of epilepsy in humans (1–3), but it is nowadays used less commonly as there are many newer drugs with a superior safety and efficacy profile available (4, 5). Phenobarbital, introduced around 1,910, was the first anti-convulsant drug replacing the use of bromide and is, in veterinary medicine, still first-choice treatment for canine epilepsy (6). Unfortunately, few alternative options remain in veterinary medicine, as the canine metabolism degrades most newer human medications preventing optimal seizures control in dogs (1). Hence the need of bromide in veterinary neurology. The use of bromide, as a possible treatment of canine epilepsy, was first described by Schwartz-Porsche et al. (7) and Podell and Fenner (8), where it proved to be an efficacious add-on therapy for refractory, phenobarbital-treated, dogs. Bromide can also be used as mono-therapy (9), with an efficacy comparable to fenobarbital (10). However the authors concluded that there were, compared with fenobarbital, several disadvantages to its use (10). Side effects are more frequent, and can include increased appetite, drinking, urinating, drowsiness and loss of activity. Further, it's half-life is long, and it can be highly variable amongst dogs, ranging from 25 days (in patients) to 46 days (in laboratory Beagles) (11, 12). Given that steady state serum concentrations are only achieved after five half-lives (13, 14), it might feasibly take approximately 125 to 230 days before stable and efficacious serum levels can be achieved. Its uptake and secretion is influenced by both dietary chloride intake and renal function (9, 11). Therefore, a clinician using bromide therapy must take into account dietary chloride intake (since a high chloride intake will tend to shorten half-life and lead to lower serum concentrations) and renal function (which decreases bromide excretion, thereby increasing circulating concentrations) (15). Although chloride intake is predominantly through ingestion of food, it is feasible that other environmental sources contribute. The ingestion of sea water will most likely influence serum levels and the same could be applicable to dogs living close to sea as air in a coastal environment has a high concentration of salt in aerosol form (16, 17). It has been reported that salt content diminishes steadily to negligible concentrations once beyond 50 km inland (16, 17). Hence, it is possible that a greater salt exposure in a coastal region could decrease bromide half-life in dogs receiving bromide, meaning that a greater dose is required to reach effective serum concentrations. The aim of the current study was to determine whether there were differences in the use of bromide as an antiseizure therapy for dogs living in coastal regions, compared with dogs living inland.

## Materials and Methods

### Study Design and Eligibility Criteria

The study is a retrospective cross-sectional study of epileptic dogs on bromide. Dogs were eligible for inclusion if they were suffering from idiopathic epilepsy as defined by Berendt et al. (18) and were being treated with potassium bromide either as monotherapy or in combination with other antiseizure medications. Further, a serum sample had to have been submitted to the University Veterinary Diagnostic Laboratory (UVDL), Utrecht, the Netherlands, for measurement of therapeutic bromide concentration. A time frame of 2 years, for the selection of cases, was chosen. Samples were either submitted by primary care veterinarians or referral specialists. Study units were defined as the individual dogs from which the samples were obtained, rather than the serum bromide concentrations (19). Therefore, when multiple samples had been submitted from the same dog, only the result of the most recent sample was used during a period when the bromide dose and food given had not been altered. Dogs were excluded if the veterinarian had used a loading dose protocol when bromide therapy was commenced, if dogs living inland had visited the coastal areas during this study period, or if their serum creatinine concentration was > the reference value used by the laboratory (i.e. reference value [in umol/L] = 60 ± the bodyweight of the dog).

### Serum Bromide Measurement

All serum bromide measurements were performed at the University Medical Center, Division Laboratory and Pharmacy, Utrecht University, Netherlands, using a slightly modified version of the gold-trichloride assay as previously described (11, 20). To a total of 500 μl of test serum 3,0 ml of 0,5% sodium chloride was added. Precipitation of proteins in the test serum was accomplished by adding 500 μl of 25% trichloroacetic acid. Samples were then centrifuged for 5 min. Two ml of supernatant was mixed with 250 μl 0 5% gold chloride and centrifuged for another 5 min. The clear supernatant was used to spectrophotometrically measure the resulting gold color at 440 nm. Quantification of bromide concentrations in this assay is based on the principle of bromide and gold chloride reacting and forming gold bromide, resulting in a specific absorption at 440 nm. The limit of quantification was 100 mg/ml and linearity was validated between 500 and 3,000 mg/l of bromide.

### Collection of Patient Information

A time frame of 2 years, during which there was only one formulation of potassium bromide available (Epikal, AST Pharma), for the selection of cases, was chosen to identify dogs eligible for inclusion. A postal questionnaire survey was sent to the veterinarians who submitted the sample, in order to gather information on the dog, and to confirm eligibility. Data obtained included: residence, type of food, diagnostic tests performed (including renal function), bromide formulation, starting date of the therapy, sampling date, bromide dose during this period, and any other treatments administered.

### Statistical Analysis

Data were analyzed with computer software IBM^®^ SPSS^®^ Statistics. Statistical significance was set at *P* < 0.05 with *P* values between 0.05 and 0.1 were considered to suggest a statistical tendency.

The main objective was to determine if there was a difference in bromide dose and bromide serum concentration in dogs living in different residences. Other factors taken into association included treatment duration, type of food, additional antiepileptic drugs (AED). The serum bromide dose was measured in mg/kg bodyweight and the bromide serum concentration was measured in mg/l serum. The treatment duration for each dog was defined as the period between the start of bromide therapy at a certain dose and sampling date. As mentioned above, no alterations in bromide dose were allowed during the treatment period. Treatment duration was converted to a binary variable with treatment duration <120 days and >120 days. The different types of food the dogs were receiving were grouped in “commercial diets”, “gluten-free antigen-limited diets”, “fresh meat diets” and “unknown/mixed diets”. Also, groups were made for concurrent therapies. The first group “None” received no concurrent therapy, another group was treated concurrently with phenobarbital, group 2 with phenytoin and group 3 received other types of medication. The different residences were determined by calculating the distance of the dog's geographic location to the closest coastal region with the “Measure distance” function in Google Maps. Dogs were grouped as follows: those living ≤ 50 km from a coastal region were assigned to group “sea side,” dogs >50 km from a coastal region were assigned to group ‘inlands. The relation between treatment period, type of food and concurrent therapies in the dogs living in the different residences were assessed using a Chi-square test. An ANCOVA was used to determine the influence of residence, concurrent therapy, treatment period and diet on serum bromide concentration and bromide dose, whilst the influence of residence on serum bromide concentration and bromide dose was assessed by an ANCOVA and a regression analysis.

## Results

### Details of Study Dogs

In the initial computer records search, a total of 658 dogs were identified that had samples submitted within the study period. Of these, postal survey results were returned for 345, of which 220 were filled in completely, enabling a dataset of 220 unique single study units to be created (Table 1). Veterinarians did not report using a loading dose protocol in any of these 220 dogs, and serum creatinine concentrations were never > the laboratory reference value.

**Table 1:** Baseline data of the study population.

**Number of study units (dogs)**	**220**
	Residence seaside: 117 (53%)
	Residence inlands: 103 (46%)
Mean bromide dose	31 mg/kg (2 mg/kg to 102 mg/kg)
Mean bromide serum concentration	1288 mg/L (352 mg/L to 3257 mg/L)

### Bromide Dose, Serum Bromide Concentrations

The mean bromide dose daily given was 31 mg/kg (2 mg/kg to 102 mg/kg), whilst the mean serum bromide concentration was 1,288 mg/L (351 mg/L to 3,257 mg/L) (Table 1).

### Residences

All study dogs lived in the Netherlands, but came from various regions. A total of 117 dogs (53%) were assigned to group seaside (<50 km from a coastal region), 103 dogs (46%) were assigned to group inlands (> 50 km from a coastal region) (Table 1).

### Treatment Duration

There was no minimum duration of treatment required. Although with a treatment period <120 days there is a risk that a steady-state bromide serum concentration has not been reached. There were no significant differences in treatment period of the dogs (>120 days or <120 days) between the two residences (*P* = 0.87). There is no significant effect of treatment duration on the bromide dose and serum bromide concentration (*P* = 0.33).

### Types of Food

**Figure 1:**
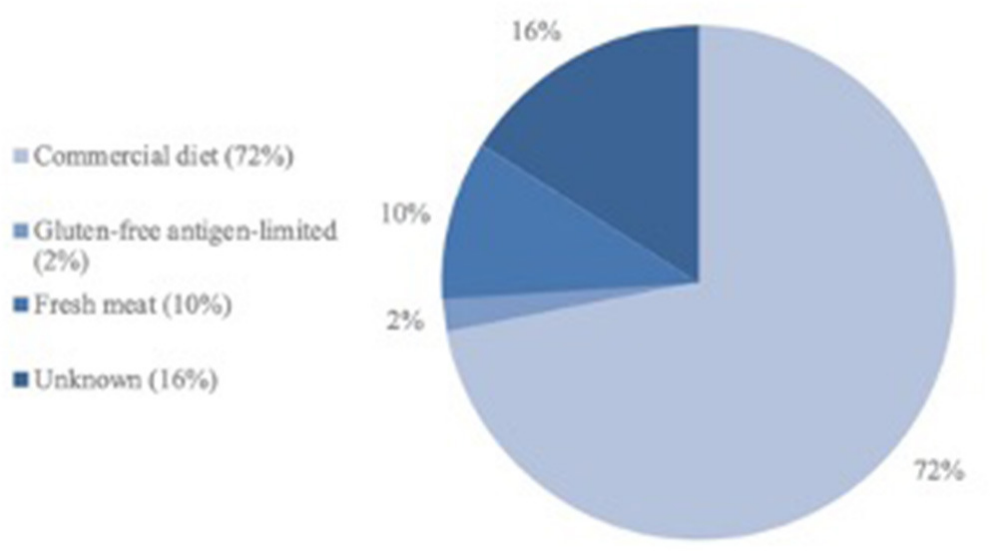
Types of food of the study population.

The dogs were fed a wide variety of types of food, with the majority (*n* = 159, 72%) receiving a variety of commercial diets from a range of manufacturers (e.g. >25). A further four dogs (2%) received a commercial gluten-free antigen-limited diet, 21 dogs (10%) received fresh meat only, whilst the type of diet was not known for the remaining 36 dogs (16%) (Figure 1). Diets were not changed between the start of treatment and sampling date. There was no difference in type of food given to the dogs between the two residences *(P* = 0.97) or influence of the types of diet on the serum bromide concentration and the bromide dose (*P* = 0.39).

### Concurrent Therapies

**Figure 2:**
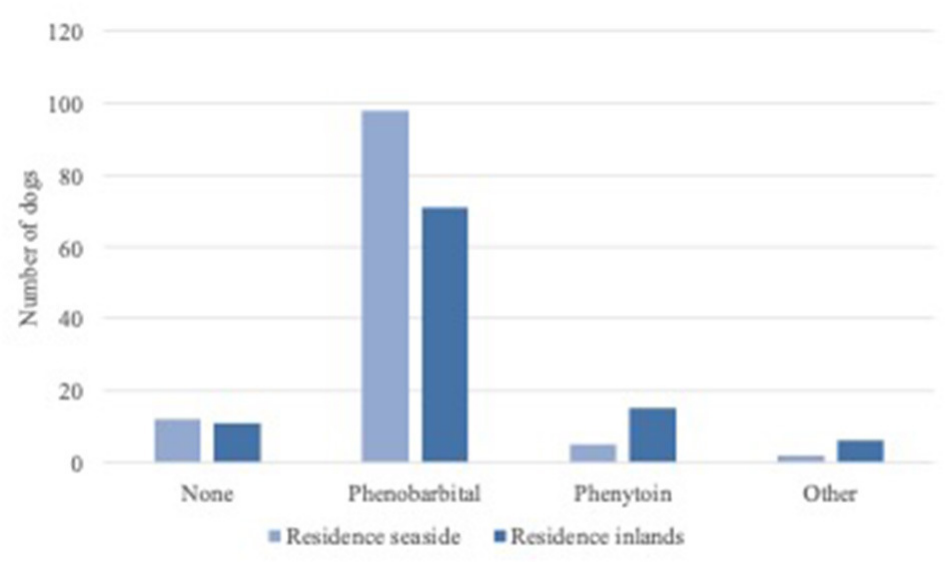
Concurrent therapies of the dogs grouped by residence.

Phenobarbital, slow-release phenytoin and levetiracetam were administered as concurrent AED. Twenty-four dogs (11%) received no concurrent therapy, where 168 dogs (76%) were treated with phenobarbital and 20 dogs (10%) with slow-release phenytoin. Eight dogs (4%) were treated with other medication such as levetiracetam, carprofen, benazepril, phenylephrine, prednisone or levothyroxine (Figure 2). Interestingly there was a significant difference in concurrent therapies between residence seaside and residence inland (*P* = 0.02). There is no significant influence of concurrent therapies on bromide dose and serum bromide concentration (*P* = 0.12).

### Effect of Residence on the Bromide Dose and Serum Bromide Concentration

**Figure 3:**
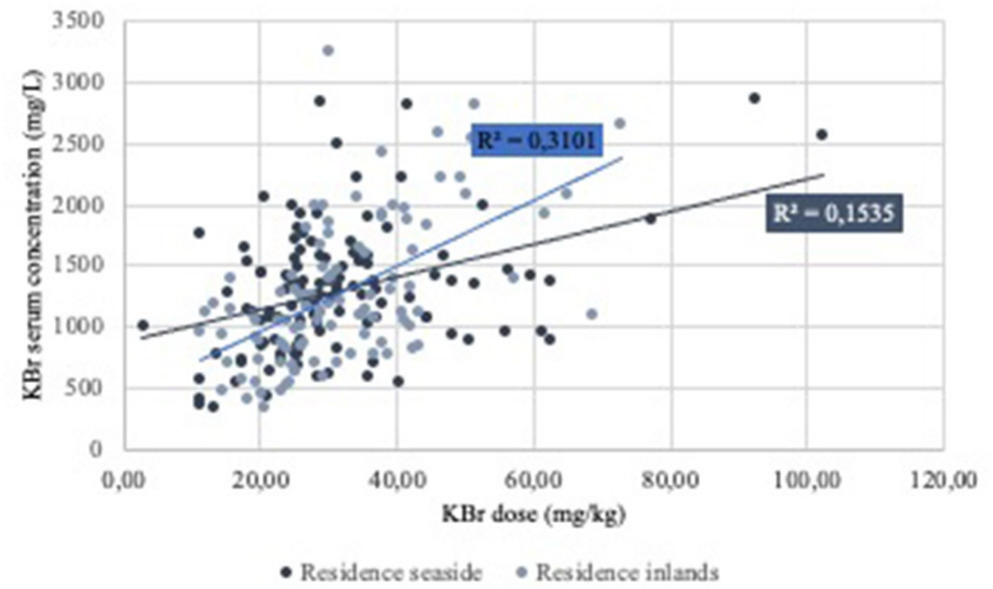
Regression analysis of the potassium bromide (KBr) dose and serum concentration of the dogs grouped by residence.

Figure 3 shows the linear regression for potassium bromide dose and serum concentration for the two residences. The regression coefficients were 0.15 for residence seaside and 0.31 for residence inlands. Both were statistically significant by itself (*P* < 0.01). This shows the need of higher doses of potassium bromide to reach a specific serum bromide concentration when living in residence seaside ompared to the other residencies. However, residence had no statistically significant influence on serum bromide concentration and bromide dose (*P* = 0.82).

## Discussion

The purpose of this study was to investigate the effect of residence on the bromide dose and serum concentration and to describe the correlation between residence, bromide dose, treatment duration, type of food, concurrent therapies and serum concentration in dogs treated with bromide. This study shows that there is a tendency for the need of higher doses of potassium bromide for dogs living close to the sea to reach an effective serum concentration, than for dogs living more inland. However, this effect was not statistically significant.

As bromide is not subject to hepatic biotransformation the only factor that can influence the bromide excretion is the amount of dietary chloride, renal function and external salt intake (9, 21). Chloride can decrease the half-life time of bromide. After the glomerular filtration, bromide will be reabsorbed in the tubules (22). The reabsorption of bromide is, compared to chloride, stronger unless high quantities of chloride are present in the tubules (11, 23). If higher quantities of chloride are present in the diet the half-life time will decrease (11). This was most likely the reason that Trepanier and Babish (11) found a significantly longer half-life time of 46 ± 9 days compared to the half-life time of 25 days found by Schwartz-Porsche and Jurgens (12). The dogs used to calculate the half-life time from Schwartz-Porsche and Jurgens (12) were fed a normal diet and the dogs of Trepanier and Babish (11) a low salt diet. For this reason, we tried to exclude the food factor as well as the renal function in this study. The exact amount of chloride was not known for most types of food, nor could be retrieved how much food they actually gave to their dogs which made it impossible to get a clear idea of the actual amount of dietary chloride given to the dog. Although all dogs were fed a wide variation of food types there was no statistically significant difference when looked at food and residence (*P* = 0.66) and a difference in dietary salt intake was therefore unlikely. And although a normal creatinine level does not exclude a decreased renal function all dogs included had creatinine levels below the reference value of the lab. So most likely the only factor that could influence the bromide serum concentration would be the residence of the dog.

There are some limitations for this study. The data we used was obtained retrospectively, which leads automatically to less controlled data collection and thus a great variation in external influences. However, the external influences considered to be important in potassium bromide therapy have been accounted for in this study by showing that there were no differences in treatment duration or type of food between the residences. Treatment duration can influence the serum concentration of bromide, when a steady state has not been reached. Steady state will be reached after five half-live times (13, 14). The half-life time of bromide is shown to be between 25 and 46 days and therefore steady state can be reached after 125–230 days (11, 12). In this study no minimum treatment duration was required, and therefore the serum concentrations of bromide might not have been stabilized yet for the patients treated <120 days. However, no significant difference in treatment duration has been found between the residences (*P* = 0.87) and therefore influence of treatment duration on the shown tendency for the need of higher potassium bromide dosages in dogs living closer to the sea was deemed low. A significant difference is found between the two residences for concurrent therapies. As can be seen in graph 2, other drugs are used in dogs living inland compared to those living closer to the sea. It is however unlikely that this finding influences the regression coefficient for potassium bromide dose and serum concentration, as no other influences on bromide absorption and excretion other than renal function and salt intake are known (9, 21). Future prospective studies with controlled study groups would be beneficial to rule out this uncertainty.

It would have been of interest to have accurate measurements of chloride levels as well but in cases treated with bromide, chloride measurement becomes with routine laboratory equipment as used in veterinary medicine impossible as it cannot differentiate properly between these two molecules. For this reason chloride is first removed from the serum sample before measuring bromide (11, 20). To measure both molecules in the same sample mass spectrometry is needed but this equipment is not routinely used in veterinary laboratories.

Based on our results dogs living close to sea likely need a higher bromide dose compared to dogs living inland. The only reason that could account for this is most likely that dogs living close to sea inhale more salty aerosols or ingest sea water through drinking (16, 17). which causes a decrease in half-life time of bromide.

## Conclusion

In this study residence, living close to sea or not, may have a potential influence on the bromide dose needed to reach an effective bromide serum concentration. Dogs that live close to see seem to need a higher amount of bromide compared to dogs living inland. This study demonstrates that if a dog is put on bromide, it is important to take into account all possible sources of sodium chloride the dog can ingest as it will decrease half-life time and hence increase the dose needed to achieve optimal serum concentrations.

## Data Availability Statement

The raw data supporting the conclusions of this article will be made available by the authors, without undue reservation.

## Author Contributions

PM was responsible for the study conception and PM and EL for data collection. Statistical analysis, data analysis, were performed by EL and JB. EL was responsible for writing. PM and BE supervised data analysis and manuscript editing. All authors contributed to the article and approved the submitted version.

## Funding

This manuscript is financially supported by the University of Utrecht, through Open Access Publishing funding.

## Conflict of Interest

The authors declare that the research was conducted in the absence of any commercial or financial relationships that could be construed as a potential conflict of interest.

## Publisher's Note

All claims expressed in this article are solely those of the authors and do not necessarily represent those of their affiliated organizations, or those of the publisher, the editors and the reviewers. Any product that may be evaluated in this article, or claim that may be made by its manufacturer, is not guaranteed or endorsed by the publisher.

## Abbreviations

UVDL: University Veterinary Diagnostic Laboratory.
